# The willingness of the Saudi Arabian population to participate in the COVID-19 vaccine trial: A case–control study

**DOI:** 10.1016/j.jtumed.2021.03.001

**Published:** 2021-03-30

**Authors:** Rania M. Felemban, Emad M. Tashkandi, Doaa K. Mohorjy

**Affiliations:** a Research Center, King Abdullah Medical City, Holy Capital, KSA; b Department of Medicine, College of Medicine, Umm AlQura University, Makkah, KSA

**Keywords:** التجارب السريرية, كوفيد-١٩, اللقاح, استعداد, التطورات العلمية, Clinical trial, COVID-19, Vaccine, Willingness, Scientific developments

## Abstract

**Objectives:**

This study examines the Saudi Arabian population's willingness to participate in clinical trials for the coronavirus disease 2019 (COVID-19) vaccine, comparing recovered cases' willingness with that of healthy volunteers.

**Methods:**

A case–control study was conducted on the Saudi Arabian population during September 2020. The data were collected from recovered COVID-19 participants as the case group, and healthy volunteers as the control group.

**Results:**

The data showed that 42.2% (n = 315) of recovered COVID-19 cases were more willing to participate in the COVID-19 vaccine trial than healthy volunteers (299; 38.1%) with a *p* < 0.001. The proportion of the participants who were willing to donate plasma was significantly higher among recovered participants, 84.2% (n = 112), than healthy volunteers, 76.3% (n = 87), with a *p* < 0.0001. The most significant factor responsible for a willingness to participate was the belief that vaccine discovery would help scientific developments (r = 0.525 and 0.465 for case and control, respectively). In comparison, significant reasons behind the unwillingness to participate were the risk of exposure to an unproven vaccine, r = 0.377 and 0.497 for case and control, respectively (p < 0.001), and a discomfort with being treated as an experimental subject (r = 0.275 and 0.374 for case and control, respectively).

**Conclusions:**

The differences in readiness toward the COVID-19 vaccine trial in our study does not indicate any passive exposure of participants to an unproven clinical trial vaccine, nor does it shed light on well-informed risk-related decisions. However, certain factors can significantly influence decision-making while contributing toward clinical research. This study's results must not be used for the individuals' recruitment bias in a COVID-19 vaccine trial.

## الملخص

**أهداف البحث:**

تهدف الدراسة إلى مقارنة رغبة الحالات المتعافية من كوفيد-١٩ والمتطوعين الأصحاء للمشاركة في التجربة السريرية للقاح كوفيد-١٩.

**طرق البحث:**

أُجريت الدراسة على السكان السعوديين خلال شهر سبتمبر ٢٠٢٠. وتم جمع البيانات من المشاركين المتعافين من كوفيد-١٩ ومن متطوعين أصحاء.

**النتائج:**

أظهرت النتائج أن ٤٢.٢٪ من الـحالات المتعافية (٣١٥) كانوا أكثر استعدادا للمشاركة في تجربة اللقاح من المتطوعين الأصحاء ٣٨.١٪ (٢٩٩). نسبة المشاركين الذين كانوا على استعداد للتبرع بالبلازما أعلى بكثير بين المشاركين المتعافين ٨٤.٢٪ (١٢٢) من المتطوعين الأصحاء ٧٦.٣٪ (٨٧). وكان أهم عامل مسؤول عن الرغبة في المشاركة هو الاعتقاد بأن اكتشاف اللقاح من شأنه أن يساعد علميا. وبالمقارنة، كانت الأسباب الهامة وراء عدم الرغبة في المشاركة هي خطر التعرض للقاح غير مثبت والشعور بمعاملة الشخص كعينة تجريبية.

**الاستنتاجات:**

لا يشير فرق استعداد المشاركين تجاه تجربة لقاح كوفيد-١٩ على أن المتعافين هم على استعداد لتلقي المخـاطر المعرضة من اللقاح التجريبي السريي غير المثبت ولا يشير إلى علم الأصحاء الجيد بالقرارات المتعلقة بالمخاطر. ومع ذلك، هناك بعض العوامل التي تؤثر بشكل كبير على اتخاذ القرار عند المشاركة في الأبحاث السريرية. وبالتالي، يجب عدم استخدام نتائج هذه الدراسة بتحيز أثناء تعيين الأفراد في تجربة لقاح كوفيد-١٩.

## Introduction

In December 2019, the world was affected by the coronavirus disease of 2019 (COVID-19), eventually declared a pandemic. It spread within months due to its high transmission but low mortality rates.^1^^,^^2^ Accordingly, healthcare facilities could not cope with the numbers, leading to a global pandemic,^1^^,^^2^ a situation that necessitated the development of a viable vaccine. Since prevention of the virus is no longer possible, there is a need for the rapid diagnosis and treatment of COVID-19 infection, so experiments on several types of antiviral drugs are being conducted.^3^ A vaccine tracker keeps track of all registered clinical trials undertaken worldwide to develop a viable COVID-19 vaccine.^4^ A more recent review in early 2020 noted that around 158 COVID-19 vaccine candidates have already been discovered and are being explored.^5^ Out of those vaccine candidates, 135 have already entered the preclinical or exploratory stage. According to US Food and Drug Administration guidelines, a potential vaccine must pass through phases of clinical trials to validate its safety and efficacy, which ordinarily takes years to complete.^5^

Several studies demonstrated the participants' willingness; in KSA, 71.5% of the participants were willing to enrol in Phase I clinical trials, while in England, 79% were willing to enrol in various preventive and therapeutic studies.^6^^,^^7^ The primary negative factor influencing the public's readiness is a lack of knowledge about the virus and the disease.^6^ Another factor is the ethical issue in speeding up the clinical trial procedure by deliberately exposing healthy individuals to disease due to the social and scientific benefits.^8^ The FDA has launched fast-track paths for vaccine availability before approval.^9^ This vaccine validation approach would facilitate high-priority outcomes and the production of required evidence for approval based on alternate efficacy markers.^9^ No studies have assessed the public's attitudes or willingness to participate in clinical trials for the COVID-19 vaccine on the Saudi population. This study examines and compares the willingness among recovered COVID-19 cases and healthy individuals to participate in a COVID-19 vaccine trial, and the factors responsible for their decision.

## Materials and Methods

### Study design

A case–control study was conducted on the Saudi population during September 2020, approved by the ethical committee. The case group contained individuals who had recovered from COVID-19 infection or who were diagnosed during the time of the study, while the control group contained normal, healthy individuals who had never been affected. The sample was obtained according to a non-probability sampling design.^8^

### Study participants

**Figure 1:**
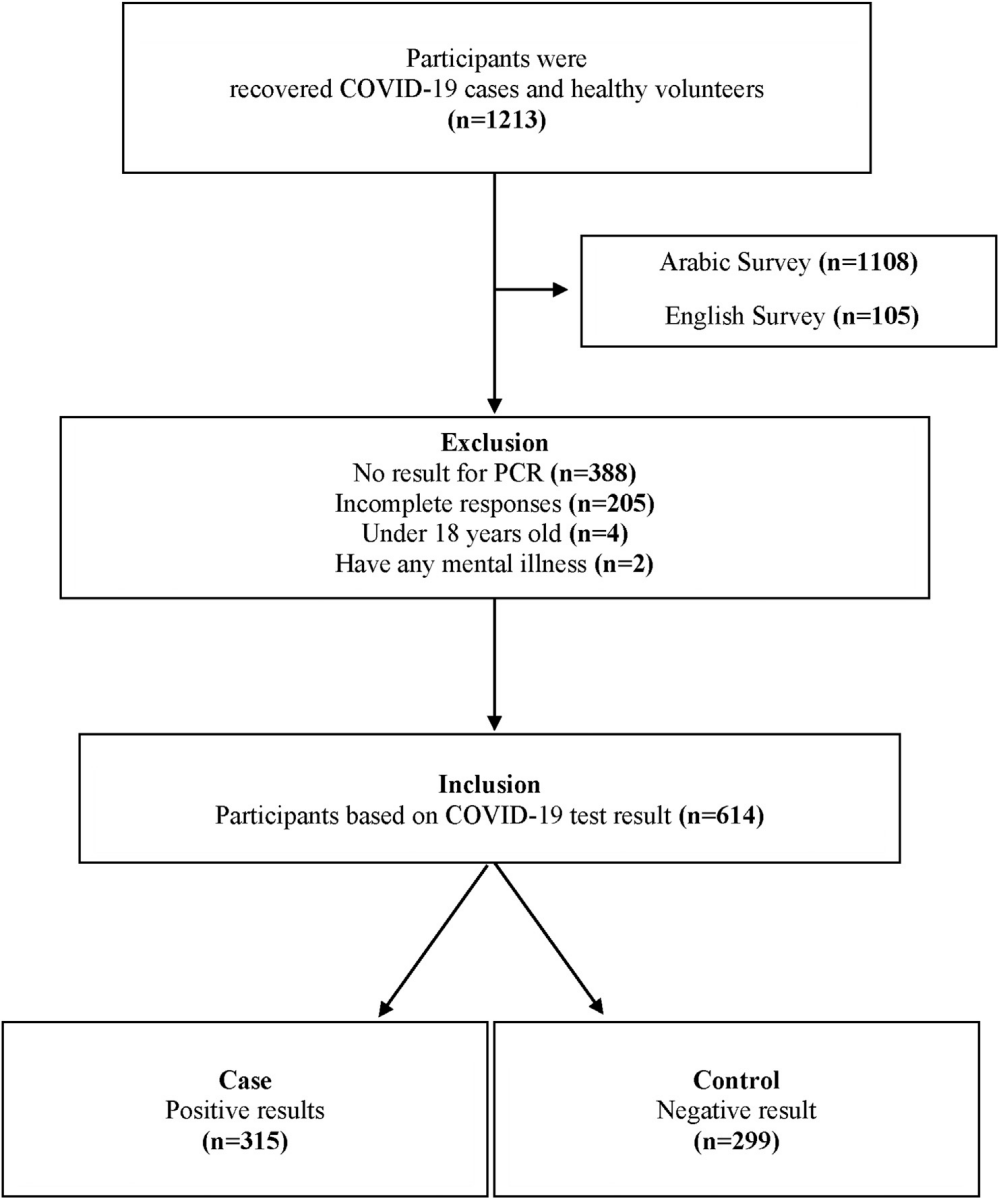
Flowchart of the recruitment process.

Initially, the recruitment was for 1213 individuals, all of whom were invited to participate in an Arabic or English survey. They contributed through a web-based link or by a phone interview. All enrolled individuals were Saudi nationals, and they were recruited irrespective of their gender, education level, and social status. Participants’ COVID-19 status was confirmed using the polymerase chain reaction (PCR) test. The study included males and females over 18, divided into two groups: case (n = 315) and control (n = 299). Later, 599 individuals were excluded from the study for failing to respond to the survey, being under 18 years of age, not having a PCR test result, or having a history of mental illness (Figure 1).

### Survey development

The survey was designed based on the literature,^10^ and it was divided into domains, without confidential information, and administered within 10 min on average. The questions were either multiple choice, a selection from many answers, or a Likert scale. The questions gathered their demographic information, included an assessment of their knowledge about clinical trials, and asked about their willingness to join a Phase I clinical trial for the COVID-19 vaccine.^11^^,^^12^ Based on the COVID-19 test result question, the case group participants were exposed to a domain that assessed their experience during the infection.

### Survey validity and reliability

Prior to the data collection phase, a pilot study was conducted among 37 participants in KSA using convenience random sampling by sharing a link on WhatsApp; they were not included in the study results. The questionnaire showed acceptable reliability (Cronbach's alpha: 93%). The following pre-testing and pilot testing methods were used to assess the questions' clarity and consistency, time of completion, and the study objectives' relativity.^10^ Five experts were engaged, followed by confirmation from a research methodologist, and a non-professional reviewed the survey's clarity and dynamics. Then, a statistician verified the overall efficacy and assessed the pilot testing result as the final stage. Minor modifications were implemented before the actual data collection.

### Statistical analysis

Data were analysed using the SPSS statistics software (version 25; IBM). The two groups' data were compared using the Student's *t*-test or the Mann Whitney *U* test, based on the data distribution and the number of groups to be compared. A chi-squared test was used to compare the intergroup differences for categorical variables. The qualitative data were represented as number and percentage, while the quantitative data were expressed using mean and standard deviation (SD) or median and inter-quartile range (IQR). For categorical variables, the *p*-value < 0.05 (assessed using a two-tailed test) was considered statistically significant.

## Results

The demographics of all the participants are shown in Table 1. A majority of the individuals in the case group were male, 31–50 years of age, Saudi citizens, married, and living with a spouse and children. A majority of the individuals in the control group were female, 30 years of age or younger, Saudi citizens, and single, and living with a relative or friend.

**Table 1:** Demographics of the Participants.

Variables	Participants (n = 614)	
	Recovered Cases (n = 315) n (%)	Healthy Volunteers (n = 299) n (%)
**Age (in years)**		
18–30	103 (32.7)	160 (53.5)
31–50	171 (54.3)	126 (42.1)
>50	41 (13)	13 (4.3)
**Gender**		
Female	156 (49.5)	194 (64.9)
Male	159 (50.5)	105 (35.1)
**Nationality**		
Saudi	233 (74)	267 (89.3)
Non-Saudi	82 (26)	32 (10.7)
**Marital status**		
Single	99 (31.4)	151 (50.5)
Married	186 (59)	138 (46.2)
Divorced	17 (5.4)	7 (2.3)
Widowed	13 (4.1)	3 (1)
**Current living situation**		
Alone	25 (7.9)	31 (10.4)
With a relative or friend	122 (38.7)	144 (48.2)
With a spouse and children	131 (41.6)	102 (34.1)
Other	37 (11.7)	22 (7.4)
**Highest level of education**		
Less than high school	16 (5.1)	4 (1.3)
High school	40 (12.7)	43 (14.4)
Bachelor's degree	167 (53)	182 (60.9)
Master's degree	62 (19.7)	57 (19.1)
PhD or higher	30 (9.5)	13 (4.3)
**Current employment status**		
Employed full-time	201 (63.8)	155 (51.8)
Employed part-time	5 (1.6)	7 (2.3)
Student	39 (12.4)	86 (28.8)
Other	70 (22.1)	51 (17)
**Monthly household income**		
Less than 5000 SAR	53 (16.8)	38 (12.7)
5000 SAR to 9999 SAR	73 (23.2)	68 (22.7)
10,000 SAR to 14,999 SAR	79 (25.1)	65 (21.7)
15,000 SAR to 19,999 SAR	43 (13.7)	57 (19.1)
More than 20,000 SAR	67 (21.3)	71 (23.7)
**Health status**		
Healthy	241 (76.5)	74 (23.5)
Unhealthy (illness other than COVID-19)	255 (85.3)	44 (14.7)

Table 2 shows the degree of willingness of both groups to participate in the clinical trial for the COVID-19 vaccine. Overall, a significantly higher proportion of the case group was willing to participate in the clinical trial than was the control group. The proportion of the case group who were either unwilling or very unwilling to participate in clinical trials was smaller than for the control group, but the difference was not significant.

**Table 2:** Degree of Willingness to Participate in the COVID-19 Vaccine Trial.

Degree	Recovered Cases (n = 315)			Healthy Volunteers (n = 299)		
	n (%)	Mean ± SD	p-value	n (%)	Mean ± SD	p-value
Very willing	52 (16.5)	3.15 ± 1.23	<0.0001	55 (18.4)	3.02 ± 1.33	.109
Willing	81 (25.7)			59 (19.7)		
Undecided	79 (25.1)			69 (23.1)		
Not willing	71 (22.5)			71 (23.7)		
Very unwilling	32 (10.2)			45 (15.1)		

The characteristics of willing participation of both groups are presented in Table 3. The data differed significantly based on age, understanding of the clinical research study terms, considering clinical research as an option for treatment, previous experience of participation in clinical research, and willingness to donate plasma as part of the research (p < 0.0001).

**Table 3:** Characteristics of Those Willing to Participate in Both Groups.

Group and Characteristics	Recovered Cases (n = 315)		Healthy Volunteers (n = 299)		p-value
	n (%)	Mean ± SD	n (%)	Mean ± SD	
**Age**					
18–30	45 (33.8)	1.80 ± .64	81 (71.1)	1.50 ± .58	**<0.0001**
31–50	76 (76)		30 (26.3)		
>50	12 (9)		3 (2.6)		
**Highest level of education**					
Less than high school	5 (3.8)	3.15 ± .94	1 (.9)	3.10 ± .74	**<0.0001**
High school	11 (8.3)		15 (13.2)		
Bachelor's degree	64 (48.1)		81 (71.1)		
Master's degree	34 (25.6)		15 (13.2)		
PhD or higher	19 (14.3)		2 (1.8)		
Understand clinical research study term	104 (78.2)	3.47 ± 1.20	91 (79.8)	3.75 ± 1.10	**<0.0001**
Consider clinical research as an option for a treatment	21 (15.8)	2.1 ± 1.10	18 (15.8)	1.86 ± 1.14	**<0.0001**
Decided or participated in a clinical research	45 (33.8)	1.80 ± .39	43 (37.7)	1.79 ± .40	**<0.0001**
Willing to donate plasma	112 (84.2)	1.48 ± .78	87 (76.3)	1.58 ± .84	**<0.0001**

Note: ∗p < .05, ∗∗p < .01, ∗∗∗p < .001.

A p-value of 5% or lower is often considered to be statistically significant.

Table 4 demonstrates the willingness to participate in clinical trials based on their experience with COVID-19 infection. Most participants who were willing to participate in the trial had experienced either severe or moderate COVID-19 infection within their family. Furthermore, a higher proportion of either home-isolated or hospitalised individuals were unwilling to participate in the trial.

**Table 4:** The Willingness of Recovered COVID-19 Cases Based on Infection Experience.

Variables		Recovered Cases (n = 315)		
		Willing n (%)	Undecided n (%)	Not willing n (%)
A family infection	Severe condition	41 (30.8)	21 (26.6)	21 (20.4)
	Moderate condition	81 (60.9)	49 (62)	65 (63.1)
	No symptoms	37 (27.8)	22 (27.8)	43 (41.7)
	Dead from COVID-19	32 (24.1)	12 (15.2)	13 (12.6)
	No one infected	19 (14.3)	10 (12.7)	15 (14.6)
Severity of infection	Home isolation	100 (75.2)	61 (77.2)	75 (72.8)
	Hospitalised	8 (6)	5 (6.3)	11 (10.7)
	ICU admission	3 (2.3)	4 (5.1)	4 (3.9)
	Required oxygen	17 (12.8)	4 (5.1)	7 (6.8)
	Other	5 (3.8)	5 (6.4)	6 (5.8)

Next, the study assessed the reasons that significantly impacted the willingness to participate in the trial. As shown in Table 5, all selected reasons were significantly associated with both groups’ willingness. Both groups had the lowest level of correlation of willingness with a diseased state or the death of a family member or a friend due to COVID-19. In contrast, the highest level of correlation was observed for the belief that vaccine discovery would help the progress of science.

**Table 5:** Spearman's Correlation Analysis of the Reasons behind Participants' Willingness.

Variables	Very willing, and willing to participate			
	Recovered Cases (n = 315)		Healthy Volunteers (n = 299)	
	*r*	*p*	*r*	*p*
Receiving public respect for the participation	.299∗∗	**<0.0001**	.341∗∗	**<0.0001**
Helping science	**.525∗∗**	**<0.0001**	**.465∗∗**	**<0.0001**
Protecting privacy and confidentiality	.445∗∗	**<0.0001**	.457∗∗	**<0.0001**
Risk awareness	.333∗∗	**<0.0001**	.334∗∗	**<0.0001**
No cost	.282∗∗	**<0.0001**	.317∗∗	**<0.0001**
Time and location convenience	.395∗∗	**<0.0001**	.484∗∗	**<0.0001**
Research activities are convenient	.362∗∗	**<0.0001**	.443∗∗	**<0.0001**
A family member or a friend suffered or deceased from COVID-19	.225∗∗	**<0.0001**	.215∗∗	**<0.0001**
Similar health condition is participating	.322∗∗	**<0.0001**	.289∗∗	**<0.0001**
Favourable opinion to participate	.269∗∗	**<0.0001**	.357∗∗	**<0.0001**
Free medical care access for complications	.296∗∗	**<0.0001**	.405∗∗	**<0.0001**

Note: ∗p < .05, ∗∗p < .01, ∗∗∗p < .001.

A p-value of 5% or lower is often considered to be statistically significant.

Table 6 assessed the correlation between the potential causes and the participants’ unwillingness to take part in the trial. In the case group, the most significant reasons for unwillingness were the risk of exposure to an unproven vaccine, a feeling of discomfort with being treated as an experimental subject, the knowledge that the vaccine would be of no help in case of the first infection, the belief that there is no risk, and the feeling that exposure to an unproven vaccine is unethical.

**Table 6:** Spearman's Correlation Analysis of the Reasons behind Participants' Unwillingness.

Variables	Very unwilling and unwilling to participate			
	Recovered Cases (n = 315)		Healthy Volunteers (n = 299)	
	*r*	*P*	*r*	*p*
Privacy and confidentiality	.073	.196	.181∗∗	**.002**
The risk of exposure to an unproven vaccine	**.377∗∗**	**<0.0001**	**.497∗∗**	**<0.0001**
No direct benefit	.098	.081	.202∗∗	**<0.0001**
Lack of knowledge of clinical research	.083	.142	.106	.066
Being treated as an experimental subject	**.275∗∗**	**<0.0001**	**.374∗∗**	**<0.0001**
Not helpful in case of the first infection	.230∗∗	**<0.0001**	.019	.745
Time commitment and location	.060	.292	.058	.316
Need permission to participate	.073	.196	.108	.061
Negative stories	.043	.448	.042	.473
The belief that there is no risk	.125∗	**.026**	-.016	.782
Exposure to an unproven vaccine is unethical	.124∗	**.028**	.211∗∗	**<0.0001**
No particular reason	-.017	.767	.993	**<0.0001**

Note: ∗p < .05, ∗∗p < .01, ∗∗∗p < .001.

A p-value of 5% or lower is often considered to be statistically significant.

## Discussion

Our study demonstrated that recovered COVID-19 cases were more willing to participate in the COVID-19 vaccine trial and donate plasma. The most significant reason behind the willingness to participate was the belief that vaccine discovery would help science progress. Factors responsible for participants’ unwillingness to participate were the risk of exposure to an unproven vaccine and discomfort with being treated as an experimental subject.

Compared to our study, most healthy individuals (69%) in the United States were willing to contribute to COVID-19 vaccine trials,^13^ compared with healthy Chinese volunteers (64.01%).^14^ In another recent Chinese study, 77.4% and 81.1% of healthy male and female participants, respectively, were willing to be vaccinated.^15^ Our study showed that only 38.1% of Saudi healthy volunteers were willing to participate in COVID-19 trials. This result is similar to the willingness of healthy Jordanian volunteers (36.1%).^16^ Based on the literature, only healthy volunteers' willingness to participate in COVID-19 vaccine trials was evaluated. Individuals who were aware of clinical trials’ concept and design exhibited a higher willingness to participate in clinical trials. Similarly, a higher proportion of individuals who had previous clinical trial experience were willing to enrol in COVID-19 clinical studies than those who have never been in any trial.^11^^,^^16^^,^^17^

Our participants from both groups were more willing to donate blood plasma. These data supported the results showing that the intervention procedure was the reason for volunteers’ unwillingness to participate in a clinical trial.^18^ Individuals are more willing to participate in clinical trials if there are less invasive activities.

Several characteristics distinguished both groups in terms of age, education, and background. In the case group, the highest proportion of willing participants belonged to the middle-aged group, while the highest percentage of the willing healthy volunteers were in the young age group. In contrast, a study reported that older individuals among all age groups showed a higher willingness to help in scientific growth and assist individuals with similar health conditions.^17^ Education and background affect the willingness to participate; the total percentage of the postgraduate case group's willingness is more than the same individuals with the same education level as the control group, indicating the positive impact of the pandemic on clinical trials' overall perception. An earlier survey revealed that a postgraduate-specialised research group had the least desire to be enrolled in clinical trials, considering their knowledge of the safety profiles and the type of investigation compared to public postgraduates.^11^

The most significant factor behind recovered COVID-19 patients' willingness is supporting science. A former study also showed that participating in clinical trials is beneficial for science.^11^ Moreover, a convenient time and location had the most significant effect in a healthy person's decision and willingness to participate in a trial, followed by a belief in helping science. In comparison, the most decisive factor determining unwillingness to participate in the COVID-19 vaccine trial is the risk of exposure to an unproven vaccine. The public is usually afraid of participating in an unknown experiment mainly due to non-scientific factors, such as spiritual or ethical beliefs, or the idea of being treated like animals.^8^

Those results have multiple strength points, starting from the research question's novelty during the pandemic and before the approval of any COVID-19 vaccine. Other strengths are the study design of a case–control with a two-tailed α (Type I error) at 0.05, and the high survey response rate of 83%. Finally, the questionnaire was validated through pre-testing and pilot methods, and showed an acceptable reliability with a Cronbach's alpha of 93%.

The limitations of this study included the selection of the population, and the type of data collection instrument. Only Saudi nationals were recruited who either never contracted or had recovered from COVID-19 infection, and who did not represent the Ministry of Health COVID-19 database. Moreover, there was a bias in the results due to the self-administration of the survey. Hence, these results could reduce the generalisability of the outcomes.

## Conclusion

Our findings highlight both groups' inclination and characteristics and readiness to participate in clinical research during such a pandemic. Despite the considerable difference between the willingness of recovered COVID-19 cases and that of healthy volunteers to contribute to a COVID-19 vaccine trial, is that neither the case group nor the fully knowledgeable control group were willing to risk exposure to an unproven clinical trial vaccine or make risk-related decisions. However, personal experiences, age, education, disease exposure, health, and clinical research illiteracy can significantly affect risk assessments and decision-making when contributing to clinical research in general. Hence, this study's results must not be used for the individuals' recruitment bias in a COVID-19 vaccine trial. Generally, the data provided an idea regarding the Saudi population's readiness to participate in such trials.

## Recommendations

Study results have implications for COVID-19 vaccine research acceptance, especially now that vaccine development has been fast-tracked. However, the high risks and the public interest of the COVID-19 vaccine trials necessitate general motivation to participate in the clinical trials. The Ministry of Health should adopt a general clinical research awareness campaign and specialised program to promote a positive vaccine uptake attitude. Focusing on the benefits for individuals, society, and science reduces their fears and minimizes clinical research illiteracy.

## Source of funding

This research did not receive any specific grant from funding agencies in the public, commercial, or not-for-profit sectors.

## Conflict of interest

The authors have no conflicts of interest to declare.

## Ethical approval

This study was approved by KAMC IRB, registered at the National BioMedical Ethics Committee, King Abdulaziz City for Science and Technology, with approval number 20–690 in August 2020.

## Authors contributions

RF wrote and designed the study, conducted research, collected data, and wrote and reviewed it. ET wrote and designed the study, conducted validation, and wrote and reviewed it. DM conducted research, conducted validation, collected data, analysed and interpreted data, and wrote and reviewed it. All authors have critically reviewed and approved the final draft and are responsible for the content and the similarity index of the manuscript.
